# Overcoming resistance to targeted therapy with immunotherapy and combination therapy for metastatic melanoma

**DOI:** 10.18632/oncotarget.18523

**Published:** 2017-06-16

**Authors:** Hilary R. Keller, Xin Zhang, Li Li, Helmut Schaider, James W. Wells

**Affiliations:** ^1^ The University of Queensland School of Medicine, Ochsner Clinical School, Brisbane, QLD, Australia; ^2^ The University of Queensland School of Medicine, Ochsner Clinical School, New Orleans, LA, USA; ^3^ The University of Queensland Diamantina Institute, Faculty of Medicine, The University of Queensland, Translational Research Institute, Brisbane, QLD, Australia; ^4^ Laboratory of Translational Cancer Research, Ochsner Clinic Foundation, New Orleans, LA, USA; ^5^ Dermatology Research Centre, The University of Queensland Diamantina Institute, Translational Research Institute, Brisbane, QLD, Australia

**Keywords:** metastatic melanoma, immunotherapy, targeted therapy, therapy resistance, immune inhibitory receptors

## Abstract

Resistance to targeted therapy is an ongoing problem for the successful treatment of Stage IV metastatic melanoma. For many patients, the use of targeted therapies, such as BRAF kinase inhibitors, were initially promising yet resistance inevitably occurred. Even after combining BRAF kinase inhibitors with MEK pathway inhibitors to offset re-activation of the MAP kinase pathway, resistance is still documented. Similarly, outcomes with immune checkpoint inhibitors as monotherapy were optimistic for some patients without relapse or progression, yet the majority of patients undergoing monotherapy have progressive disease. Will immunotherapy and combination therapy trials overcome resistance in metastatic melanoma? In an effort to treat resistant disease, new clinical trials evaluating the combination of immunotherapy with other therapies, such as kinase inhibitors, adoptive cell therapy, chimeric CD40 ligand to boost costimulation, or a tumor-specific oncolytic virus enhancing granulocyte macrophage colony-stimulating factor (GM-CSF) expression, are currently underway. Updated studies on the mechanisms of resistance, immune escape and options to reinvigorate immune cells support the continued discovery of new and improved forms of therapy.

## INTRODUCTION

Despite the high cure rates associated with the early diagnosis and removal of melanoma, patients with Stage IV metastatic disease have a 5-year survival expectancy of approximately 18% [1–3]. Melanoma thus accounts for the majority of deaths related to skin cancer. Davies et al. reported that approximately 37–59% of melanomas contain a mutation in the gene that encodes *BRAF* [4]*,* which was associated with younger age at diagnosis and poorer survival [5, 6]. In recent years, drugs that target molecularly defined vulnerabilities (such as BRAF kinase) in human melanoma have been clinically validated as effective melanoma therapies [7]. Nearly all patients, however, experience a relapse of the disease due to the emergence of acquired drug resistance [8]. Resistance to therapy has now become a major obstacle for successful melanoma treatment. Efforts to overcome drug resistance with combination BRAF/MEK kinase inhibitors or monotherapy with immune checkpoint inhibitors have, so far, only prolonged time to progressive disease [9–14]. Here, we discuss the current views on the mechanisms of resistance, immunotherapy to overcome T cell dysfunction, and options to reinvigorate T cells, such as adoptive cell therapy.

### Resistance to targeted therapy

Drug resistance is defined by either progression of disease or locoregional recurrence despite treatment, and further includes the appearance of new lesions [15, 16]. Resistance to BRAF inhibitor therapy is likely due to either a pre-existing intrinsic mechanism, or an acquired mechanism [14, 17–19], the latter being the case for most patients who initially respond but later progress [17]. Pre-existing intrinsic mechanisms of resistance primarily occur due to genetic alterations, mutations, loss of function, or overexpression of genes involved in either the PI3K/AKT signaling pathway or the MAPK/ERK pathway [8, 17, 18, 20–26]. The PI3K/AKT pathway involves the activation of mTOR, eventually resulting in reduced apoptosis, promoting growth, protein synthesis, and proliferation [17]. The MAPK/ERK pathway involves a cascade of activation and phosphorylation by kinases, promoting the proliferation, survival, and differentiation of cells [17]. The most common cause of acquired resistance is due to the reactivation of the MAPK/ERK pathway [8, 14, 17, 27–29], followed by the upregulation of the PI3K/AKT pathway [14]. Another potential mechanism of kinase inhibitor therapy is the activation of the noncanonical Hedgehog pathway observed in melanoma cell lines [30, 31]. The treatment of resistant cells to a specific Hedgehog pathway inhibitor, Gant61, observed in one study, restored sensitivity to the BRAF kinase inhibitor, vemurafenib [30]. Of note, however, tumor heterogeneity, i.e. the same patient tumor with more than one mechanism of resistance detected [14], may contribute to the persistence of disease progression despite combination kinase inhibitor therapy, and presents a new challenge against resistance.

Recently, we observed that acquired multidrug resistance is caused by a stress response induced by chemotherapy such as docetaxel, the BRAF kinase inhibitors vemurafenib and dabrafenib, the MEK inhibitor trametinib, hypoxia, or low nutrient environments [32]. The stress response involves chromatin remodeling and activation of signaling cascades and is characterized by an increase in expression of the nerve growth factor receptor CD271 [32], a proposed marker of melanoma stem cells [33], and one that may suppress CD8^+^ T cell function [34]. The stressed state is further signified by the loss of melanoma differentiation markers such as Melan-A and tyrosinase [32]. The loss of tumor-associated target antigens, which are normally expressed on the surface of melanoma cells, may prevent recognition by melanoma-specific cytotoxic T cells [34]. Tumor specific T cells are a key component of immune defense against melanoma, and impaired antigen recognition results in an inability of the immune system to control tumor growth.

### Immunotherapy to overcome resistance

In an effort to address and combat resistance to monotherapy, current treatments for metastatic melanoma either combine the BRAF kinase inhibitor dabrafenib with the MEK inhibitor trametinib or recommend the monoclonal antibody, ipilimumab, against the immune checkpoint inhibitor cytotoxic T-lymphocyte antigen 4, CTLA-4, for first-line therapy [17, 35, 36]. Combined targeted therapy with two kinase inhibitors is indicated for unresectable or metastatic melanoma [17], showing improved overall survival in combination compared to the BRAF inhibitor alone [37]. However, resistance to combined BRAF/MEK kinase therapy is still described [13, 38]. Further studies and ongoing trials suggest that combination immunotherapy, pairing ipilimumab with the programmed cell death 1, PD-1, monoclonal antibody, nivolumab, prolongs progression-free survival in untreated metastatic melanoma patients compared to either nivolumab alone [39] or ipilimumab alone [39–41]. However, an increased incidence of severe drug-related adverse events was observed in over half of those patients undergoing combination therapy [39–41], including diarrhea, fatigue, pruritis, rash, nausea, colitis and fever. Many were reversible with immunosuppressants but some patients required additional systemic glucocorticoids, infliximab, or mycophenolate immunosuppressive therapy instead.

CTLA-4 blockade toxicity or therapeutic response may be related to gut-resident bacteria, according to recent studies [42]. One study found that T cell responses specific for *Bacteroides* species within the gut were associated with anti-CTLA-4 efficacy both in mice and in patients [43]. This effect was eliminated in mice treated with antibiotics to eradicate *Bacteroides* from the gut and reversed when *Bacteroides* was replaced by gavage. Furthermore, the study found that melanoma patients treated with CTLA-4 blockade grew gut-specific *Bacteroides* with antitumor characteristics. Another recent study evaluating the influence of the intestinal microbiome on immunotherapy-related colitis found that increased *Bacteroides* representation within the gut correlated with resistance to anti-CTLA-4-induced colitis [44]. Together, these studies may indicate an adjunct to combination immunotherapy with promoting, establishing, or replenishing favorable gut microbiota to both induce anti-tumor activity and prevent therapy-mediated toxicity.

Furthermore, ipilimumab has shown improved overall survival in previously untreated or treated and refractory metastatic disease [35, 36]. The antibody target, CTLA-4 or CD152, is an immune inhibitory marker expressed on T cells after prolonged T cell activation and is constitutively expressed on memory CD4^+^ and CD8^+^ T cells as well as regulatory T cells [45, 46]. CTLA-4 competes with CD28 costimulation necessary for T cell activation, and thus suppresses the activation and further proliferation of T cells, resulting in a diminished immune response [45–48]. A study evaluating disease progression after ipilimumab therapy examined differences in the expression of immune antigens and candidate genes in patient melanoma tumor samples both pre and post-therapy [49]. The authors found that the expression of a tyrosine kinase cell cycle regulator, *TTK*, and the expression of a gene encoding the antiapoptotic protein survivin, *BIRC5*, both increased in tumors that progressed despite therapy. However, the expression of immune antigens, e.g. CD3, CD4, CD8, and CTLA-4, remained unchanged a year after ipilimumab therapy. While promising, the study was limited by a sample size of four patients. As part of the selection criteria to help elucidate mechanisms of ipilimumab resistance, the patient pool was narrowed down to those who developed some form of an autoimmune reaction to the therapy [49].

More recently, in an effort to increase the efficacy of ipilimumab monotherapy, a clinical trial combining the CTLA-4 inhibitor with an oncolytic virus derived from herpes simplex virus 1 (HSV-1) was developed, revealing a tolerable safety profile and greater efficacy in combination than with either alone [50]. An ongoing Phase III trial is currently evaluating the combination of pembrolizumab, the PD-1 inhibitor, with or without the same oncolytic HSV (NCT02263508). An oncolytic virus is one that selectively infects, replicates within, and kills tumor cells, allowing further dissemination of the virus throughout the tumor tissue [51]. Talimogene laherparepvec or T-VEC, the oncolytic HSV, recently underwent optimistic safety and efficacy trials in single-arm and comparable interventional studies of T-VEC monotherapy [52]. The oncolytic virus was modified by the deletion of two specific herpes simplex 1 viral genes, the neurovirulence factor *ICP34.5* and the *ICP47* gene, to evade the host’s anti-viral response thus allowing the virus to replicate within tumor cells, and to subsequently lyse them [51, 53–57]. The deletion of the *ICP47* gene in particular is thought to induce a systemic tumor-specific immunity by deleting an inhibitor of antigen presentation, therefore rendering the tumor cells vulnerable to an immune response [58–61]. The virus was further modified by the insertion of a gene encoding the expression of human granulocyte macrophage colony-stimulating factor, GM-CSF, to promote anti-tumor immune responses by recruiting and activating antigen-presenting cells, APCs [62].

Phase I and II T-VEC trials revealed replication of the oncolytic virus within tumor cells, the expression of GM-CSF, and an overall response rate of 26% in the intralesional T-VEC group compared to 6% in the group given subcutaneous GM-CSF [63–65]. The Phase III trial revealed a higher durable response rate and longer overall survival in Stage IIIB, IIIC, or IV patients [52], likely due to the systemic tumor-specific immunity induced by the virus [58, 66]. Furthermore, T-VEC was determined to be safe and well tolerated with only minor viral prodromal adverse effects – fever, chills, myalgias, and mild skin site reactions [52, 63, 64]. While promising as monotherapy, the view that combination therapy to target different mechanisms of action – immune checkpoint inhibitors with oncolytic viruses enhancing the expression of pro-immune factors like GM-CSF as well as inducing systemic anti-tumor immunity–may provide more durable and efficacious response rates long term, as current trials suggest.

### Immune exhaustion and dysfunction in the tumor microenvironment

Clinically, compared to ipilimumab, patients treated with monoclonal antibodies directed against PD-1, such as nivolumab and pembrolizumab, appear to have higher response rates, sustained tumor regression, and may be better tolerated [17, 67, 68]. Despite differences in clinical outcomes, both PD-1 and CTLA-4 are inhibitory markers expressed in T cell exhaustion–a dysfunctional state in response to persistent antigen stimulation and inflammation–along with other markers such as lymphocyte-activation gene 3 (Lag-3), and T cell immunoglobulin domain and mucin domain 3 (Tim-3) [69–71]. Both Lag-3 and Tim-3 are expressed on T cells, regulatory T cells, B cells, dendritic cells, NK cells, and NKT cells [72]. However, Lag-3 acts as a CD4 homolog by binding MHC class II [72–74], while Tim-3 is involved in T cell tolerance [72, 75]. Although much of the research on T cell exhaustion was done studying chronic viral infection, recent studies suggest that the tumor microenvironment in metastatic melanoma involves infiltrating lymphocytes (TILs) expressing characteristics of exhaustion – namely, PD-1 expression on T cells [69, 76, 77]. In examining the effector function of these PD-1^+^ TILs, Fourcade et al. showed that T cell function is impaired in those infiltrating CD8^+^ T cells or cytotoxic T lymphocytes (CTLs) that express PD-1 and more so in PD-1^+^ TIM-3^+^ infiltrating CTLs than single positive cells [78]. TILs that co-express many immune inhibitory markers are thus more dysfunctional [72, 79, 80]. This study, however, assessed CTL function by IFNγ, TNFα, or IL-2 cytokine release, but failed to show direct cytotoxic activity via tumor cell lysis.

Interestingly, in the exhausted state, CTLs may exhibit residual IFNγ production, albeit at low levels, but may express high levels of granzyme B with some residual cytotoxic capacity, although these studies were conducted by examining chronic viral infection, not the tumor microenvironment [69, 81, 82]. Spranger et al. showed that the PD-1 ligand, PD-L1, is expressed on melanoma cells with TILs, and indicates that IFNγ may increase PD-L1 expression; however, this study was performed in mice [83, 84]. These findings were validated in humans, where both melanoma tumor cells and infiltrating monocytes were shown to express PD-L1 in association with TILs in PD-L1^+^ melanomas [85]. This study also revealed the association of PD-L1 expression and TILs with IFNγ expression, which a prior human tumor cell line study revealed as an inducer of PD-L1 expression [86]. In a mouse model of tamoxifen-inducible liver cancer, tumor-specific CD8^+^ T cells, or TST cells, were determined to be dysfunctional early during tumorigenesis, suggesting that T cell dysfunction in later stages of cancer may be established during the initial tumor formation [87]. Furthermore, the study suggested that TST cell dysfunction was initially reversible but became fixed later on, was likely induced by persistent antigen exposure, and appears distinct although similar to exhausted T cells from chronic viral infection [87]. In another recent study evaluating the metabolic effects of T cell exhaustion in virus-infected mice, increased PD-1 signaling was associated with impaired glucose uptake and metabolism that occurred early during CD8^+^ T cell exhaustion [88]. This metabolic dysfunction was suggested to have been partially inhibited by PD-1 signaling [88]. While the mechanism of exhausted TILs within the tumor microenvironment appears to be variable and unclear, likely the functional impairment of exhausted TILs is reversible but requires further study [69].

### Reinvigorate T cell function – adoptive T cell therapy

In addition to assessing the mechanism of exhausted or dysfunctional TILs, determining ways in which to reverse and reinvigorate the activity of TILs specific to the tumor, without promoting autoimmunity is necessary, especially if prognostic indicators for robust immunotherapy or targeted kinase therapy response cannot be clearly elucidated. The most recent trial involving adoptive cell therapy (ACT) revealed no benefit with the addition of total body irradiation given to those following the lymphodepleting chemotherapy regimen in preparation for ACT [89]. These ACT studies have been chronicled to summarily include lymphodepleting chemotherapy, administering *ex vivo* activated and expanded TILs, and high dose IL-2, resulting in a complete response rate of 24% in metastatic melanoma patients and an overall 3-year survival rate of 51% [90–99]. Interestingly, a pilot trial evaluating the co-administration of vemurafenib with ACT in metastatic melanoma patients revealed a relatively similar safety profile as either treatment alone, and favorable clinical responses [100]. Although the trial involved a small cohort of eleven patients, all of whom received both vemurafenib and ACT, the majority of the patients achieved a partial response, yet only two patients had complete regression. Further studies are needed to evaluate combination therapy involving different mechanisms of action, which may prove to be more synergistic than either alone.

To support the synergistic effect of combining ACT with other treatment modalities, in particular BRAF or MEK kinase inhibitors, prior studies have revealed that BRAF kinase inhibition may enhance T cell recognition by increasing expression of melanocyte differentiation antigens without impairing lymphocyte viability or function [101, 102]. Similarly, MEK inhibition has been shown to increase tumor antigen expression, but may impair lymphocyte proliferation and function without affecting viability [103]. While ACT remains a promising and evolving therapy that is personalized and patient-specific, predictive markers or factors that may predispose patient tumor cells to acquire resistance to the current therapy – BRAF and MEK kinase inhibitors–continues to demand investigation. Likely mechanisms to be explored include increased CD271 and PD-L1 expression in response to aberrant IFNγ release, thereby downregulating differentiation markers and impairing CTL activity, or alternatively, kinase signaling pathway reactivation–either through MAPK or PI3K pathways.

### Potential biomarkers of resistance

Markers predictive of resistance, recurrence, and prevention of resistance would effectively redirect treatment towards combination therapy as first-line, ideally for patients in previously untreated but invasive disease, and not limited to those with advanced or unresectable melanoma. New biomarkers may even be used as new targets for combination therapy. A recent study evaluating potential biomarkers for response to treatment was conducted in metastatic melanoma patients treated with anti-CTLA-4 followed by anti-PD-1 therapy, using tumor biopsies at specific time points before and during treatment [104]. Response to therapy was defined as either with no radiographic evidence, stable disease, or decreased tumor volume, using the Response Evaluation Criteria in Solid Tumors (RECIST) criteria [16, 104]. The study demonstrated a higher density of CD8^+^ T cells present early during treatment in those tumors that responded to anti-CTLA-4, despite the lack of biomarkers clearly identified prior to treatment [104], although a prior study has shown a higher number of PD-1 and PD-L1 expressing cells too [105]. In those that responded to anti-PD-1 therapy, they found an increased expression of CD8, CD4, CD3, PD-1, PD-L1, and Lag3 early during treatment [104]. While it has been previously published that anti-CTLA-4 or PD-1 monotherapy induces infiltration by TILs [105–108], this study suggests that CD8^+^ T cells infiltrating the tumor before therapy are particularly correlated with tumor response during therapy. In conjunction with a prior study evaluating response to anti-PD-1 therapy [105], CD8^+^ T cell density may be useful in predicting response to either anti-PD-1 [104, 105, 108] or CTLA-4 therapy [107], and reduced expression of CD8, PD-1, and PD-L1 despite therapy may correlate with disease progression [105].

**Figure 1:**
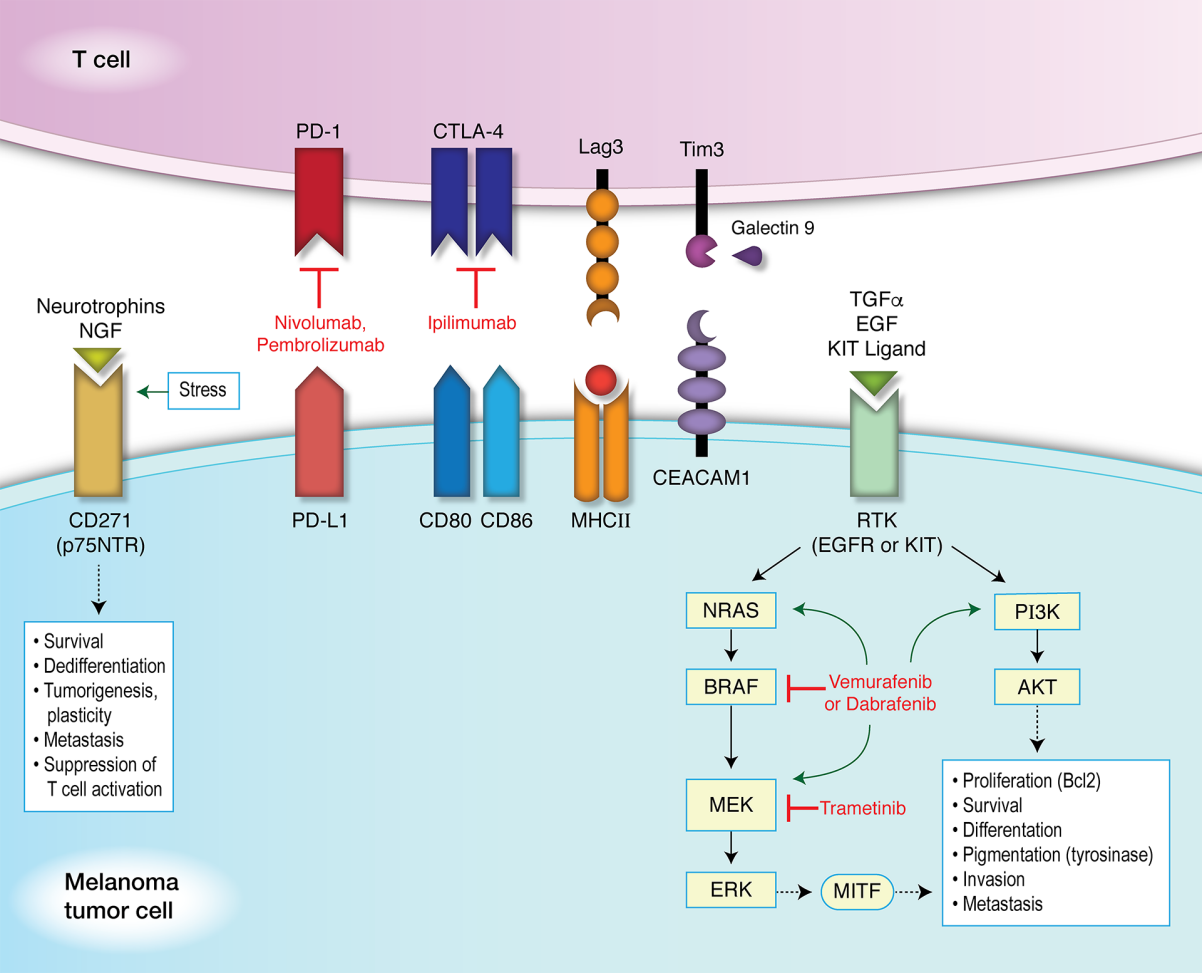
Targeted and immunotherapy against immune inhibitory receptors and resistance mechanisms Immune inhibitory receptors and their ligands are paired as shown. Neurotrophins and nerve growth factors (NGF) bind to CD271 (p75NTR), whose expression can be induced under conditions of stress, resulting in downstream signaling pathways promoting survival, dedifferentiation, tumorigenesis, plasticity, metastasis, and the suppression of T cell activation. Transforming growth factor-α (TGFα), epidermal growth factor (EGF), and KIT ligand (stem cell factor or STEEL) can bind to a receptor tyrosine kinase (RTK), such as the EGF receptor (EGFR) or KIT receptor (c-KIT receptor or CD117). This promotes the activation of either the MAPK pathway or PI3K/AKT pathway. Kinase inhibitors can induce mutations in components of either pathway, as shown by green arrows. These induced mutations then promote the activation of downstream signaling, bypassing the targeted inhibition and resulting in the phosphorylation of microphthalmia-associated transcription factor (MITF). Expression of MITF leads to differentiation and pigmentation (via tyrosinase activity) as well as the proliferation and survival of melanocytes (through the upregulation of Bcl2). Activation of these pathways also promotes invasion and metastasis in melanoma tumor cells. Tim3 (HAVCR2) forms a heterodimer with CEACAM1 (carcinoembryonic antigen-related cell adhesion molecule) inducing T cell inhibition, and also binds its ligand, Galectin 9 to then suppress T helper cell type 1 (Th1) function and induce cell death.

Ideally, a panel of biomarkers to indicate T cell dysfunction within the tumor microenvironment, or markers on melanoma cells specific to therapy resistance would be best for analyzing these states while accounting for variations in any one marker alone. These may include markers examining the T cell exhaustive state on TILs, such as CTLA-4, PD-1, TIM-3, and LAG-3. Markers on melanoma cells include PD-L1 and CD271 (Figure 1). The limitations to using a panel of biomarkers include the method by which to determine the expression of these markers efficiently. These markers are not ubiquitously expressed in tumor tissue or TILs, and may require larger samples to identify isolated areas of expression. Furthermore, while a panel of markers may be able to expand the number of possible markers expressed in a given area of tumor tissue, CD8^+^ T cells are likely to be in close proximity to the PD-1^+^ and corresponding PD-L1^+^ expressing cells in both the tumor and invasive margin [105]. This may likely extend to other proposed biomarkers involved in immune inhibition, exhaustion, or dysfunction as well.

### Future immunotherapy combinations

To add to the current and ongoing research into potential biomarkers to predict responsiveness to therapy and mechanisms of response to therapy, mechanisms and predictors of resistance must also be considered. Treatment approaches that target different aspects of the “cancer-immunity cycle” [109] which may be dysfunctional are promising. These targets include factors within the tumor microenvironment that may modulate the T cell immune response against the tumor, or immune checkpoint inhibitors expressed on either T cells or tumor cells that may contribute to both resistance and T cell dysfunction in combination [110, 111]. New and effective therapy combinations are likely to be elucidated in the many ongoing clinical trials (Table 1). These studies either preemptively prevent resistance by combining a kinase inhibitor with immune inhibitor therapy or treat refractory patients with therapy tailored to the tumor’s resistance mechanism. A few trials evaluating combination immunotherapy and targeted kinase therapy include treating with ipilimumab, pembrolizumab, or an anti-PD-L1 antibody with BRAF and MEK kinase inhibitors. The BRAF kinase inhibitor vemurafenib is also being evaluated with adoptive cell therapy in one trial, while another is pairing vemurafenib with an anti-PD-L1 antibody. Two studies are currently recruiting those who relapse or progress. One study is examining progression after BRAF kinase therapy then specifically tailoring subsequent therapy to the tumor’s resistance mechanism, either with a cyclin dependent kinase inhibitor, fibroblast growth factor receptor (FGFR) inhibitor, PI3K inhibitor, or c-MET inhibitor (NCT02159066). The other study is comparing ipilimumab with or without nivolumab in those who relapsed after anti-PD-1 therapy. Another two studies are soon to begin recruiting patients to boost the costimulatory interaction between CD40 on APCs and CD40 ligand (CD40L) on activated T cells. One study will evaluate the effect of a CD40 agonist with pembrolizumab. The other study will use an adenovirus encoding a chimeric CD40L that is injected into the tumor directly. Interestingly, a Phase I trial is currently recruiting metastatic melanoma patients for treatment with patient T cells electroporated with RNA to express c-Met specific chimeric antigen receptors. More ongoing trials include pembrolizumab with or without T-VEC, ACT with or without pembrolizumab, indoximod (the indoleamine 2,3-dioxygenase inhibitor with a role in controlling inflammation and T cell tolerance [112]) with either pembrolizumab, ipilimumab, or nivolumab, and many others [17].

**Table 1:** Completed and active clinical trials for combination therapy in metastatic melanoma*

Regimen	Phase	Status	Sponsor	NCT identifier
Ipilimumab and Dabrafenib +/− Trametinib	I	Completed	GSK	NCT01767454
Durvalumab (anti-PD-L1) and Trametinib +/− Dabrafenib	I	Active, not recruiting	Med Immune	NCT02027961
Pembrolizumab + Dabrafenib + Trametinib vs Pembrolizumab + Dabrafenib or Trametinib	I/II	Recruiting	Merck	NCT02130466
Nivolumab + Trametinib +/− Dabrafenib	II	Recruiting	MD Anderson	NCT02910700
Atezolizumab (anti-PD-L1) and Vemurafenib +/− Cobimetinib (MEK inhibitor)	I	Active, not recruiting	Genentech	NCT01656642
Atezolizumab (anti-PD-L1) +/− Vemurafenib and Cobimetinib (MEK inhibitor)	III	Recruiting	Hoffmann-La Roche	NCT02908672
Encorafenib (RAF inhibitor) and Binimetinib (MEK inhibitor) until progression then + Ribociclib (CDK4/6 inhibitor) or FGFR inhibitor or PI3K inhibitor or Capmatinib (c-MET inhibitor)	II	Active, not recruiting	Array BioPharma	NCT02159066
Ipilimumab +/− Nivolumab after progression or relapse post-anti-PD-1	II	Recruiting	Memorial Sloan Kettering	NCT02731729
Vemurafenib + ACT	I	Terminated	NCI	NCT01585415
ACT +/− Pembrolizumab	II	Recruiting	NCI	NCT02621021
ACT using autologous T cells with retrovirally transduced TCR	I	Recruiting	Loyola University	NCT02870244
Pembrolizumab +/− T-VEC	III	Recruiting	Amgen	NCT02263508
IDO inhibitor and Ipilimumab or Pembrolizumab or Nivolumab	I/II	Recruiting	NewLink Genetics	NCT02073123
Pembrolizumab +/− Epacadostat (IDO inhibitor)	III	Recruiting	Incyte	NCT02752074
CD40 agonist with Pembrolizumab	I/II	Recruiting begins May 2017	MD Anderson	NCT02706353
Pembrolizumab + Intratumoral adenovirus encoding chimeric CD40L	I/II	Not yet recruiting	MD Anderson	NCT02719015
Autologous T cells expressing c-MET chimeric antigen receptors	I	Recruiting	University of Pennsylvania	NCT03060356

*NCT = ClinicalTrials.gov number; Refs = references associated with study; GSK = GlaxoSmithKline; ACT = adoptive cell therapy; NCI = National Cancer Institute; TCR = T cell receptor; T-VEC = Talimogene laherparepvec; IDO = indoleamine 2,3-dioxygenase; CDK4/6 = cyclin-dependent kinases 4 and 6; FGFR = fibroblast growth factor receptor; PI3K = phosphatidyl-inositol-3 kinase; c-MET = tyrosine protein kinase Met or hepatocyte growth factor receptor encoded by MET gene [119].

### Concluding remarks

The treatment of patients with metastatic melanoma has thus experienced huge progress with the use of molecular targeted inhibitors and immunotherapy. Recently published results of the Sunbelt Melanoma Trial revealed no survival benefit for adjuvant high dose interferon in those with a single positive sentinel lymph node (Stage III), whether they underwent complete lymph node dissection alone or with interferon therapy [113]. Despite the mixed and controversial results of studies evaluating the efficacy of high dose interferon therapy as a method to induce an effective immune response [114–118], the current trend towards combination immunotherapy appears much more promising. While targeted inhibitors can be effective initially, many patients relapse within one year. Immunotherapy, in the form of antibodies against specific tumor antigens to promote immune recognition and response, shows sustained progression-free survival and even complete recovery in many patients. Mechanisms of resistance in those patients who do not respond to drug therapy, or who respond but later progress, are largely unknown but are of utmost importance to reveal in order to define better treatment strategies for optimal patient-specific precision medicine.
